# Renin-angiotensin-aldosterone system polymorphisms: a role or a hole in occurrence and long-term prognosis of acute myocardial infarction at young age

**DOI:** 10.1186/1471-2350-8-27

**Published:** 2007-05-22

**Authors:** Erica Franco, Luigi Palumbo, Francesca Crobu, Matteo Anselmino, Simone Frea, Giuseppe Matullo, Alberto Piazza, Gian Paolo Trevi, Serena Bergerone

**Affiliations:** 1 Department of Internal Medicine, Cardiology Division, San Giovanni Battista Hospital, University of Turin, Italy; 2 Department of Genetics, Biology and Biochemistry, University of Turin, Italy

## Abstract

**Background:**

The renin-angiotensin-aldosterone system (RAAS) is involved in the cardiovascular homeostasis as shown by previous studies reporting a positive association between specific RAAS genotypes and an increased risk of myocardial infarction. Anyhow the prognostic role in a long-term follow-up has not been yet investigated.

Aim of the study was to evaluate the influence of the most studied RAAS genetic Single Nucleotide Polymorphisms (SNPs) on the occurrence and the long-term prognosis of acute myocardial infarction (AMI) at young age in an Italian population.

**Methods:**

The study population consisted of 201 patients and 201 controls, matched for age and sex (mean age 40 ± 4 years; 90.5% males). The most frequent conventional risk factors were smoke (p < 0.001), family history for coronary artery diseases (p < 0.001), hypercholesterolemia (p = 0.001) and hypertension (p = 0.002). The tested genetic polymorphisms were angiotensin converting enzyme insertion/deletion (ACE I/D), angiotensin II type 1 receptor (AGTR1) A1166C and aldosterone synthase (CYP11B2) C-344T. Considering a long-term follow-up (9 ± 4 years) we compared genetic polymorphisms of patients with and without events (cardiac death, myocardial infarction, revascularization procedures).

**Results:**

We found a borderline significant association of occurrence of AMI with the ACE D/I polymorphism (DD genotype, 42% in cases vs 31% in controls; p = 0.056). DD genotype remained statistically involved in the incidence of AMI also after adjustment for clinical confounders.

On the other hand, during the 9-year follow-up (65 events, including 13 deaths) we found a role concerning the AGTR1: the AC heterozygous resulted more represented in the event group (p = 0.016) even if not independent from clinical confounders. Anyhow the Kaplan-Meier event free curves seem to confirm the unfavourable role of this polymorphism.

**Conclusion:**

Polymorphisms in RAAS genes can be important in the onset of a first AMI in young patients (ACE, CYP11B2 polymorphisms), but not in the disease progression after a long follow-up period. Larger collaborative studies are needed to confirm these results.

## Background

Five to ten percent of new acute myocardial infarction (AMI) occur in individuals younger than 45 years [1]. An Italian population registry (GISSI study) [2] reported a similar, even if slightly lower, incidence (about 2%) in our country at the end of the XXI century. At this age, AMI is characterized by low mortality rates, less extensive coronary artery disease (CAD), better residual left ventricular function and a favourable prognosis in short and medium follow-up [3].

As known, CAD is a multifactorial disease influenced by environmental and genetic factors, in fact, in a younger population smoking, dyslipidemia and familiarity are more frequent than in older patients [2-4].

Although the role of these environmental factors in the development of AMI has been clearly established, the role of non-conventional risk factors remains undefined. In the last few years a great interest has been focused on genetic factors with the intent to find common markers that could identify a subgroup of patients at higher risk of death or with a worse prognosis in which new therapeutic timings and interventions could be tested.

Particular interest has been focused on the renin-angiotensin-aldosterone system (RAAS) [5,6] in view of its physiological role and of the established benefits of ACE inhibitors therapy.

In the enzymatic cascade, angiotensinogen (AGT) is cleaved by renin to produce angiotensin I, which is further converted in the bioactive octapeptide angiotensin II (ATII) through the action of angiotensin I converting enzyme (ACE), a membrane-bound, zinc metallo-endopeptidase involved in the metabolism of many small peptides. The cellular effects of ATII in human adults are mainly mediated by the angiotensin II type 1 receptor (AGTR1). Aldosterone synthase (CYP11B2), sensible to the effects of ATII, catalyses the final step of aldosterone biosyntesis in adrenal glomerulosa. Because of the various physiologic effects of ATII, including vasoconstriction, promotion of vascular smooth muscle cells growth and increase of extracellular collagen matrix synthesis, genetic variation of the level of each RAAS component could probably affect a wide variety of clinical phenotypes.

AGTR1 is known to play a pivotal role in the physiopatology of several cardiovascular abnormalities [7-10], and ACE and AGT are important in blood pressure homeostasis [11,12], so it is not surprising that the genes coding for members of the RAAS and its products are being investigated in relation with AMI.

Aim of this study is to investigate the effects of ACE insertion/deletion (ACE I/D), AGT1R A1166C and CYP11B2 C-344T polymorphisms on the occurrence and the long-term prognosis of AMI at young age in an Italian population.

## Methods

### Patients

The study population was recruited from patients aged ≤ 45 years, consecutively admitted between 1992 and 2002 to the Coronary Care Unit of the University Cardiology Division of St. Giovanni Battista Hospital, Turin, who satisfied the WHO criteria for diagnosis of acute myocardial infarction [13].

Risk factors data were obtained from each patient including smoking habits [14], hypercholesterolemia [15,16], diabetes status [16], and hypertension [17].

A positive family history was considered if the patient had a first-degree relative with CAD at the age < 55 years for men or at < 65 years for women.

Patients with known congenital hypercoagulable status, with declared cocaine abuse or with a known disease limiting life expectance were excluded.

Each patient was matched with a healthy control subject, recruited by General Practitioners, by sex, age (± 1 year) and diabetes status. Control subjects were recruited in general practitioners out patient' clinics. Exclusion criteria for control cases were CAD and any known cardiomyopathy, neoplasm, a severe illness limiting life expectance or refusing consent.

The population recruited consisted of 201 patients and 201 matched controls.

Follow-up consisted in investigating new hospital admissions, performing periodic ambulatory visits and telephone interviews; follow-up was completed in 183 patients (91% of the starting population study) up to year 2005. The percentage of cases lost at follow-up is presumably due to the long follow-up period (9 ± 4 years) related to undetectable register variations.

Endpoint events considered were death, new AMI, and revascularisation procedures (angioplasty or coronary artery bypass graft surgery).

The study was performed according to the principles of the Declaration of Helsinki, and informed consent was obtained from each patient.

### DNA genotyping

Genomic DNA was extracted from 200 μl of peripheral blood lymphocytes according to a standard salting-out method [18].

ACE: the 287 bp insertion/deletion (I/D) polymorphism in intron 16 of the ACE gene was determined according to the method of Zhu [19]. PCR products of 190 bp for the D allele and 490 bp for the I allele were resolved on a 2% agarose/0.5× TBE gel by ethidium bromide staining.

AGTR1: the A/C transversion at base 1166 in the 3' untranslated region of the AT1R gene was determined as described previously [20]. A 349 bp PCR product was digested with DdeI for 2 hours at 37°C and resolved on a 2% agarose/0.5× TBE gel by ethidium bromide staining. After restriction digest, the 1166C allele was cut into 139 and 210 bp fragments.

CYP11B2: The T/C transversion 344 base before the start codon of the CYP11B2 gene was determined as previously described [21]. The 273 bp PCR fragment represents the wild-type T allele. After HaeIII digestion at 37°C, the fragments were cut in a 202 bp (and little PCR fragments) if the C allele occurred. Fragment length was resolved on a 2% agarose/0.5× TBE gel and then ethidium bromide staining.

### Statistical analysis

Allele and genotype frequencies of the analyzed polymorphisms were calculated by direct gene counting; a chi-square test was used to test for the Hardy-Weinberg. Each polymorphism and each risk factor, both in the acute and follow-up phase, are expressed as percentages of the respective strata and compared between patient strata by means of the Pearson and Maximum-Likelihood Chi square test (p value ≤ 0.05 considered significant).

In order to control for confounding variables, that may have influenced occurrence and long-term prognosis of AMI, multiple logistic regression analyses were performed based on statistically relevant differences in gender and conventional risk factors (smoking habits, family history, hypercholesterolemia and hypertension) distributions between both patients and controls and patients with and without events. Among conventional risk factors, only those more represented in CAD patients (p value ≤ 0.05) were included in the multivariate analysis. The best subset models were run applying odds ratio (OR) likelihood scores.

The time course from the first AMI event to the considered end-point (cardiac death, new myocardial infarction, revascularization procedures) was analysed by the Kaplan-Meier method.

All the analysis performed using STATISTICA (StatSoft Inc, Tulsa, Oklahoma, USA, version 7.1).

## Results

The study population consisted in 201 patients and 201 controls (mean age 40 ± 4 years, range 25–45 years).

Male patients were 182 (90.5%), the most frequent risk factors (Table 1) were cigarette smoking (72.9% in cases vs 36.5% in controls, p < 0.001) and familial history of CAD (58.6% vs 9.9%, p < 0.001). Significant differences were observed also for hypercolesterolemia (52.7% vs 20.2%, p = 0.001) and for hypertension (31.5% vs 7.4%, p = 0.002).

**Table 1:** Conventional risk factors.

	Smoke	FamiCAD^a^	Hypertension	Hypercol^b^
Control N (%)	74 (36.5)	20 (9.9)	15 (7.4)	41 (20.2)
Case N (%)	148 (72.9)	119 (58.6)	64 (31.5)	107 (52.7)
P value*	<10^-4^	<10^-4^	0.002	0.001

* Pearson and M-L Chi-square; ^a^FamiCAD: family history for CAD; ^b^Hypercol: hypercholesterolemia.

The allele and genotype frequencies are described in details in Table 2.

**Table 2:** Genotype distribution in patients and in healthy controls.

		**Case (%)**	**Control (%)**	**Chi square p value**	P value multiple regression*	**OR for determinant polymorphisms**
**ACE I/D**	DD	84 (42%)	63 (31%)	**0.056**	**0.005**	**2.39 (1.25–4.58) p = 0.008**
	**ID**	88 (44%)	96 (48%)			
	**II**	29 (14%)	42 (21%)			
**AGT1R**	**AA**	100 (50%)	117 (58%)	0.231	0.214	
	**AC**	84 (42%)	69 (34%)			
	**CC**	17 (8%)	15 (7%)			
**CYP11B2**	**CT**	87 (43%)	97 (48%)	**0.054**	0.978	
	**CC**	55 (27%)	35 (17%)			
	**TT**	59 (29%)	69 (34%)			

Total		201 (100%)	201 (100%)			

* Corrected for gender, smoke, family history for CAD, hypertension and hypercholesterolemia.

Genotype frequencies in the overall sample fit the Hardy-Weiberg equilibrium and were similar to those found in other studies on Caucasian population.

We found a statistically significant association between AMI and ACE polymorphism. The DD genotype alone, reaching borderline significance in the univariate analysis (42% in cases vs 31% in controls; p = 0.056) remained significant also after adjustment for conventional risk factors (p = 0.005) with a independent role on predicting events (OR 2.39 CI 1.25–4.58, p = 0.008); whereas the CYP11B2 polymorphism (CC genotype, 27% vs 17%, p = 0.054) reached borderline significance only in the crude analysis, but lost an independent role after adjustment for confounders.

On the other hand, during the 9-year follow-up (65 events, including 13 deaths) we demonstrated a statistically significant difference between patients with or without events concerning the AGTR1 (Table 3), in which the heterozygous AC genotype resulted more represented in the event group (p = 0.016).

**Table 3:** Genotype distribution according to follow-up events.

		**Events (%)**	**No events (%)**	**Chi square p value**	P value multiple regression*	**OR for determinant polymorphisms**
**ACE I/D**	DD	25 (38%)	53 (45%)	0.252	0.191	
	**ID**	27 (42%)	52 (44%)			
	**II**	13 (20%)	13 (11%)			
**AGT1R**	**AA**	26 (40%)	68 (58%)	**0.016**	0.308	**0.48 (0.24–0.96) p = 0.038**
	**AC**	35 (54%)	38 (32%)			
	**CC**	4 (6%)	12 (10%)			
**CYP11B2**	**CT**	27 (42%)	52 (44%)	0.679	0.849	
	**CC**	21 (32%)	31 (26%)			
	**TT**	17 (26%)	35 (30%)			

Total		65 (100%)	118 (100%)			

* Corrected for gender, smoke, family history for CAD, hypertension, hypercholesterolemia.

**Figure 1:**
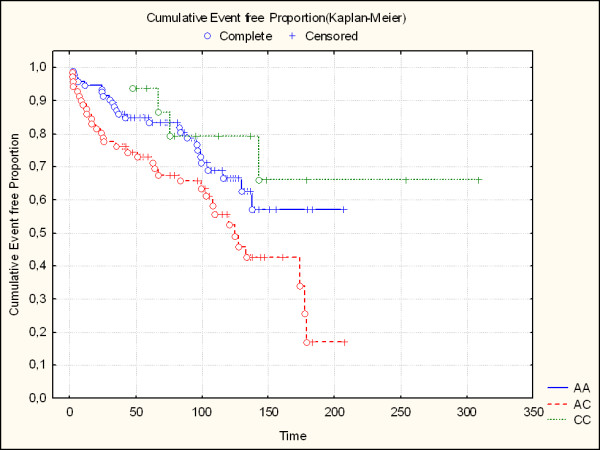
Kaplan Meier curves for AGT1R polymorphisms.

The worst prognosis of patients with the AC genotype is successively confirmed by the Kaplan-Meier event free curves (Figure 1) considering the time course from the first AMI event to the different end-points (cardiac death, myocardial infarction, revascularization procedures).

Furthermore in the multiple logistic regression analyses the heterozygous AC genotype resulted as a protective polymorphisms on the onset of events at follow-up also when correcting for conventional risk factors (OR 0.48 CI 0.24–0.96, p = 0.038). Instead prevalence of DD homozygous genotype of ACE and CC genotype of CYP11B2 did not diverge enough in the two groups to reach statistical significance.

## Discussion and conclusion

Epidemiological and clinical data from studies performed on young adults with premature AMI have shown that the occurrence of AMI in young Italian people is uncommon (2% in the GISSI study) [1,2] and that the prognosis in short-term outcome is good (mortality rate lower than 2%). For the risk factors profile, young patients have significantly higher rates of smoking, dyslipidemia and family history for CAD, compared with older patients [2]. A promising but inherently difficult area of study is the identification of genes that predispose to CAD.

The ECTIM study [11] first demonstrated that the homozygote form of a deletion polymorphism of ACE could be an independent risk factor for AMI.

In the current study, we compared the distributions of conventional risk factors and RAAS polymorphisms (ACE, AGTR1, CYP11B2) in a population of young patients with AMI during the hospital recovery for acute event and after a long-term follow-up.

ACE is expressed on the surface of the cell in many tissues (particularly in lungs), but also circulates in a soluble form obtained by the cleavage of the extracellular portion from the endothelial cells [22]. The analyzed polymorphism is a 287 base pair Alu insertion/deletion (I/D) in intron 16 of ACE gene in chromosome 17; DD genotype is associated with increased serum and cellular concentrations of ACE, that results in enhanced convertion of angiotensin I in angiotensin II [19].

Cambien [5] first demonstrated a possible role of DD genotype as cardiovascular risk factor; most of the subsequent studies have shown no significant effects of this polymorphism on the extent of CAD but mainly on the onset of acute coronary syndrome, data suggesting a possible role in the mechanism involving plaque instability, ulceration and thrombosis [22].

In a recent study Palmer et al. [23] conclude that ACE I/D genotype may provide additional prognosis information in patients with AMI, suggesting an association with DD genotype and mortality after cardiac events that was excluded in previous studies [24,25].

In our work we support the role of DD as a relevant risk factor in the acute phase, with an independent role also when adjusting for conventional risk factors. This result is of high interest if thought as a new identifiable and modifiable (ACE inhibitors, since the DD genotype enhances cellular concentration of ACE) risk factor in specific subgroups (family history for CAD) of patients. Conversely, accordingly to previous literature data [24,25], we did not find any association between DD and mortality or other events (new AMI, revascularization procedures, angioplasty or coronary bypass graft surgery) after AMI.

AGTR1 is expressed mainly in vascular smooth muscle cells and in the myocardium. A polymorphism in the 3' untranslated region of AGTR1 gene was identified [11], corresponding to an A→C transversion at nucleotide position 1166 of the mRNA sequence.

An association between AGTR1-C allele and a modified response of the receptor to angiotensin II [5] has been suggested, thus justifying the reported statistically significant correlation of AGT1R-CC with increased cardiovascular risk, together with ACE DD polymorphism [5,6], in a synergistic effect.

However, Andrikopoulos et al. [10] found that there was no significant association among patients with the A1166C polymorphism in the AGTR1 gene and increased mortality for AMI.

Our study, instead, after a long-term follow-up shows an unfavourable role of AC genotype after the acute coronary event; however, this result needs to be confirmed in larger studies.

The human aldosterone synthase (CYP11B2) gene is located on chromosome 8 and the polymorphism was studied in the promoter region of the gene at nucleotide 344 from the translation start site, where the residue could be cytosine or thymidine.

The activity of the CYP11B2 gene is primarily regulated by the renin-angiotensin system through the actions of angiotensin II.

This polymorphism has been correlated to increased circulating levels of aldosterone, thus influencing arterial hypertension, cardiac fibrosis and, consequently, both diastolic dysfunction and ventricular remodelling evolution after AMI [26].

Kupari et al observed, in young Finnish subjects, that the CC genotype of CYP11B2 polymorphism is a strong predictor of left ventricular diameters, mass and ventricular filling fraction.

It has been showed that smoking and dislypidemia are more potent risk factors for nonfatal MI in males who have the C allele of CYP11B2 [27].

In the present study, we found a statistically significant association between CYP11B2 polymorphisms and AMI, suggesting a possible role as cardiac risk factor, but not independently from the conventional ones.

In conclusion our work, although if based on a limited sample size, has the potential of a long follow-up period after the first cardiac event. Our results show that polymorphisms in RAAS genes could be important in the onset of a first AMI event in young patients (ACE, CYP11B2 polymorphisms) as in the disease progression.

Many contradictory results have been published on RAAS polymorphisms in the attempt to find out new associations with clinical-therapeutic features or biochemical determinations.

In a multifactorial disease like CAD, in which the weight of conventional risk factors is so high especially in young people, and in which genetic profile is so heterogeneous due to many low penetrant candidate genes possibly involved, the clinical usefulness of genetic findings needs to be carefully discussed.

The role of different polymorphisms in individual risk assessment for primary and secondary prevention programs is strictly linked to the possible interaction with many known conventional risk factors.

## Competing interests

The author(s) declare that they have no competing interests.

## Authors' contributions

EF partecipated in the design of the study, in the clinical evaluation and selection of patients, in the follow-up interviews, drafted the manuscript; LP partecipated in the design of the study, in the clinical evaluation and selection of patients, in the follow-up interviews, in the selection of healthy controls, drafted the manuscript; FC carried out the immunoessay, partecipated in the sequence allignment, revised the manuscript; MA partecipated in the statistical analysis, in the clinical evaluation of patients, drafted the manuscript; S.F partecipated in the clinical evaluation of patients, in the follow-up interviews; GM partecipated in the design of the study, carried out the immunoessay, partecipated in the sequence allignment, revised the manuscript; AP partecipated in the design of the study, carried out the immunoessay, partecipated in the sequence allignment, revised the manuscript; GPT.: partecipated in the design of the study, in the clinical evaluation of patients, revised the manuscript; SB partecipated in the design of the study, in the clinical evaluation of patients, in the follow-up interviews, drafted the manuscript.

## Pre-publication history

The pre-publication history for this paper can be accessed here:


